# Increased fecundity of *Aphis fabae* on *Vicia faba* plants following seed or leaf inoculation with the entomopathogenic fungus *Beauveria bassiana*

**DOI:** 10.1371/journal.pone.0223616

**Published:** 2019-10-07

**Authors:** Rasmus Emil Jensen, Annie Enkegaard, Tove Steenberg

**Affiliations:** Department of Agroecology, Section of Plant Pathology and Entomology, Aarhus University, Flakkebjerg, Denmark; University of Vienna, AUSTRIA

## Abstract

Since the discovery that entomopathogenic fungi can live inside plants as endophytes, researchers have been trying to understand how this affects mainly plants and herbivores. We studied how inoculation of *Vicia faba L*. (Fabales: Fabaceae) plants with *Beauveria bassiana* (Balsamo-Crivelli) Vuillemin (Ascomycota: Hypocreales) (strain GHA) either via the seeds or leaves influenced the nymph production of two successive generations of *Aphis fabae* Scopoli (Hemiptera: Aphididae). While we did not find any difference in nymph production for the first generation of aphids, second-generation aphids on both seed- and spray inoculated plants produced significantly higher numbers of nymphs than aphids on uninoculated plants. This emphasizes the importance of two (or multi-) generational experimentation. *Beauveria bassiana* was recovered from 26.0, 68.8 and 6.3% of respectively seed-, spray inoculated and control plants, thus, demonstrating its ability to live as an endophyte in *V*. *faba*. The confirmation that plants inoculated with entomopathogenic fungi can have a positive effect on pest insects makes careful consideration of these multi-trophic interactions imperative.

## Introduction

Today we know that plants associate with a wide array of mutualistic symbiotic microorganisms, which play a vital role for plants, as they act as the first line of defense against invading pathogens [1], provide nutrients [2], and protect plants from abiotic stress [3, 4]. One group among these microbes, the entomopathogenic fungi, has previously escaped the attention of scientists. Interest in entomopathogenic fungi as plant symbionts started in the 1990s [5], and has increased significantly in the last ten years, as the multifunctional lifestyle and the ecological role of these fungi outside their arthropod hosts is being unraveled [6]. Newly discovered roles of entomopathogenic fungi include endophytism [5, 7], plant disease antagonism [8], plant growth promotion, and rhizosphere colonization [9, 10].

The fact that the entomopathogenic fungus *Beauveria bassiana* (Balsamo-Crivelli) Vuillemin (Ascomycota: Hypocreales), an already widely used biocontrol agent, can live inside plant tissue as an endophyte has created interest in investigating how the endophytic lifestyle of entomopathogenic fungi can be used to improve their efficacy and environmental persistence as biocontrol agents [10, 11, 12, 13]. *Beauveria bassiana* has been found to colonize a wide array of important crop species such as maize, potato, date palm, banana [11], soy bean [14], tomato [15], and cassava [16]. It has also been shown that endophytic *B*. *bassiana* can adversely affect arthropod herbivores [17] exemplified by reduced longevity of the tomato leaf miner feeding on tomato plants [18], prolonged development time and reduced fecundity of the green peach aphid on sweet pepper [19], as well as increased juvenile mortality of the cotton leaf worm feeding on bread wheat [20]. Several researchers have, however, also found neutral effects on herbivores [13, 21].

Results of endophytic *B*. *bassiana* having a negative effect on herbivores rarely focus on more than one generation [21], and as insect immunity is influenced by successive exposures to the same pathogen, observing longer-term effects on pest insects, when possible, is important [22, 23]. Thus it is for instance well documented that sub-lethal doses of pesticides can stimulate aphid reproduction (hormesis) [24, 25] and that the effect can be enhanced in transgenerational experimentation [26].

The mechanisms behind negative effects on herbivores by endophytic entomopathogenic fungi have been suggested to be caused by an upregulation of the plants' defense and/or fungal metabolites that could be transported through the plants' vascular tissue and, thereby, affecting herbivores directly [10]. We know from another group of fungal plant symbionts, the mycorrhizal fungi, that they can induce systemic resistance (ISR) against pathogens [27, 28].

This study aimed to investigate how inoculation via seeds or leaves of faba bean *Vicia faba L*. (Fabales: Fabaceae) plants with *B*. *bassiana* affects the fecundity of two consecutive generations of the black bean aphid *Aphis fabae* Scopoli (Hemiptera: Aphididae) in a clip-cage experiment on whole plants. We hypothesized that colonization with *B*. *bassiana* would have a detrimental effect on the herbivore fecundity. The fecundity of two generations of *A*. *fabae* was studied, i.e. nymphs of the first generation were followed until adulthood, and then their own production of offspring was studied under the same conditions as the first generation.

## Materials and methods

A clip-cage experiment with *A*. *fabae* on *V*. *faba* plants inoculated with *B*. *bassiana* was conducted four times (twice in winter 2017/2018 and twice in spring 2018). The experiment consisted of three treatments: plants inoculated with *B*. *bassiana* by seed treatment, plants where leaves were sprayed with a *B*. *bassiana* spore suspension, and non-fungus-inoculated control plants. Plants were kept in climate chambers in a blocked arrangement by treatment throughout the experiments. Plants were checked for endophytic growth of *B*. *bassiana* after experimentation.

### The fungus

*Beauveria bassiana* (strain GHA reisolated from the BotaniGard® product) was propagated on potato dextrose agar (PDA) medium and harvested after 2–3 weeks (20°C, darkness) in a 0.1% Tween20 solution by scraping the surface of the fungus culture with an inoculation spreader (Sarstedt A/S). The preparation of the spore suspension was achieved by, first, centrifuging the collected fungal material twice at 4.000 rpm (3076 x g) for 4 minutes and then adjusting the concentration to 10^7^ spores/ml by adding 0.1% Tween20 solution after counting spores in a Fuchs-Rosenthal counting chamber. Germination percentage was checked the following day by observing >200 spores to ensure it was adequate (above 90%).

### Preparation of plants

Prior to sowing, *V*. *faba* seeds (cv. ‘Columbo’) were surface disinfected by soaking for two minutes in 70% ethanol, rinsing in sterile water, submersion for two minutes in 1% sodium hypochlorite and rinsing three times in sterile water. One mL of the final rinse water was pipetted and plated on PDA medium with an inoculation spreader to assess the surface disinfection efficacy. After surface disinfection of the seeds, one third were soaked in 40 ml spore suspension (10^7^ spores/ml) of *B*. *bassiana* for two hours. Control seeds and seeds for spray inoculation were soaked in 0.1% Tween20 for two hours. Three to four seeds were then sown in 0.5l pots in soil (1:1:1 peat:soil:sand) that had been autoclaved for 25 minutes at 121°C. Plants were thinned to one plant per pot after the emergence of seedlings. The plants were kept in net covered cages [height x width x depth; 85cm x 65cm x 75cm] in a greenhouse at 20°C and 70% RH with the photoperiod supplemented with light to L:D 16:8 in the dark season. The plants received water and nutrients two times daily via an automatic irrigation system. After three weeks, plants were used in the experiment. A second batch of plants (same method as above) were prepared one week later for the second generation of the fecundity experiments. Two hours prior to the addition of aphids, the plants were sprayed with either a 10^7^ spore/ml suspension of *B*. *bassiana* (leaf spray treatment) or a 0.1% Tween20 solution (seed inoculation and control) with a 40ml “Travel set” sprayer (produced for Zebra A/S). Two first true leaves of plants were sprayed until run-off (approximately two pushes on the small pump spray). Spraying of individual plants with the spore suspension was performed in a fume hood with no risk of cross contamination.

### The aphids

**Fig 1:**
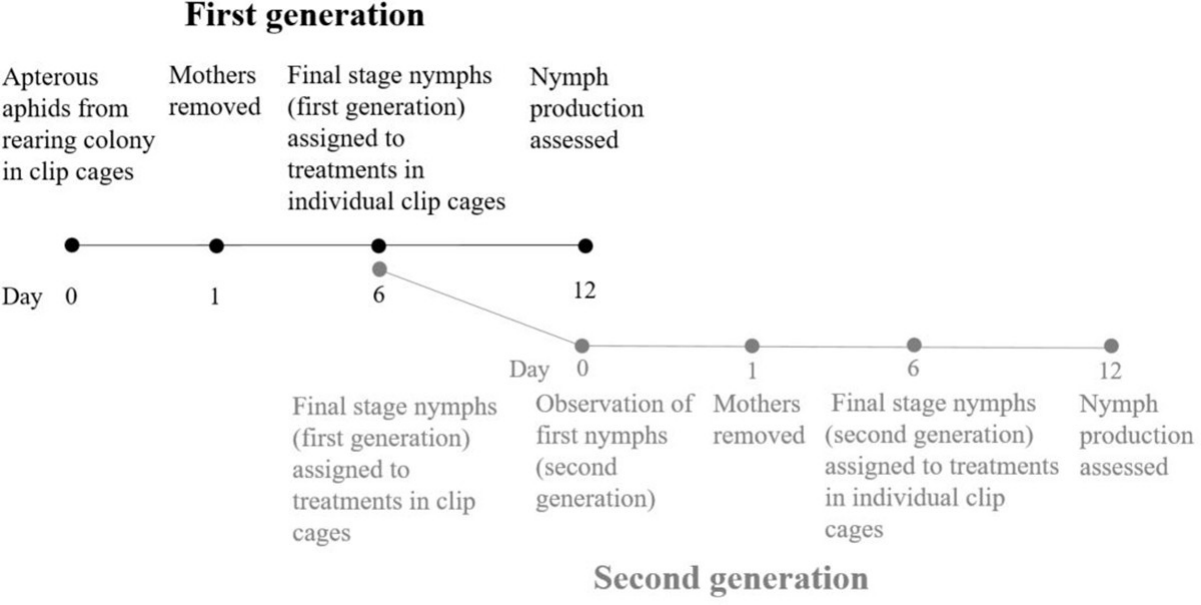
Diagram of the experiment showing key time points.

A rearing colony of *Aphis fabae* was kept more than a year on *V*. *faba* cv. Columbo plants in a cage in a greenhouse at 20°C, 70% RH and 16:8 L:D. To initiate the uniform cohort of aphids, four to six apterous adults were placed in a single clip cage (total 12–15 clip cages) on two to three weeks old non-treated *V*. *faba* plants placed in cages at the abiotic conditions mentioned above (Fig 1). Clip cages were composed of two rings (ca 3mm) cut from an acryl round tube (25 mm inner diameter) glued to a metal hair clip, cushioned with sponge material on the inside for a tight close, and a fine mesh net on the outside. After 24 hours, the adults were removed, and the nymphs produced during the 24 hours were allowed to develop for five days to become fourth stage nymphs to be used in experiments.

### Fecundity experiment

#### First-generation aphid mothers

Fourth stage nymphs were assigned to the following treatments: 1) *V*. *faba* plants inoculated with *B*. *bassiana* through seeds, 2) plants sprayed with a *B*. *bassiana* spore suspension and 3) control plants (prepared as described in ‘*Preparation of plants’*) (Fig 1). Nymphs were placed individually in clip-cages (i.e. one nymphs per clip cage) on nine plants per treatment–these nymphs were destined to develop into the first-generation mothers of the fecundity experiment. Clip cages were positioned on the first two true leaves (same leaves that were sprayed) of each plant, with one clip cage per leaf providing a total of 72 subsequent aphid mothers for the first generation fecundity experiment, including all four repetitions. All plants were placed in climate chambers at 20°C, 70% RH and a 16:8 L:D regime (5,000 lux).

First-generation mothers were allowed to produce nymphs for six days before the number produced was assessed. It was also assessed whether the adult was dead, missing, or had developed into an alate. Alate formation, which can be a stress response, is important to consider as alates produce less offspring. Dead adults were surface disinfected and placed on moist filter paper in a petri dish to check for mycosis.

#### Second-generation aphid mothers

Parallel to the first generation experiment, 1 to 3 fourth stage nymphs from the initial uniform aphid cohort were put in clip-cages on three plants per treatment (same batch of plants as first-generation mothers) (Fig 1). They were checked daily for reaching adulthood and initiation of nymph production. Once the first nymph was observed, nymph production was allowed to continue for 24 hours before the mothers were removed to ensure a uniform cohort of nymphs. These nymphs were destined to develop into second-generation mothers and were allowed to develop together for five days in clip-cages, after which they were placed in individual clip-cages as described for the first generation on plants of similar treatments (second batch of plants). We obtained 58, 60 and 39 second-generation mothers for seed, leaf and control treatment, respectively, including all four repetitions. The second-generation mothers were allowed to produce nymphs for six days in the same manner as described for the first generation. After six days, their nymph production and status was assessed as described above.

### Re-isolation of the fungus

After removal of the aphids from the fecundity experiment, *V*. *faba* plants used in both first- and second-generation experiments were checked for colonization by *B*. *bassiana* by placing plant material on PDA medium with 25ppm Novobiocin to re-isolate *B*. *bassiana*. After harvest of plants, roots were cleaned in tap water with detergents and plants were cut into 2 leaf pieces (2 first true leaves), 2 stem pieces (6 cm long) (just above the soil surface and mid-height of plant, respectively) and 2 root pieces (6 cm long) (from the primary root). Plant material was then rubbed thoroughly in tap water with detergent and subsequently surface disinfected in a flow hood by being submerged for two minutes in 70% ethanol, rinsed in sterile water, and submerged for two minutes in 1% sodium hypochlorite followed by three rinses in sterile water. One mL of the final rinse water was pipetted and plated on PDA medium with an inoculation spreader to assess the surface disinfection efficacy. After surface-disinfection, the root and stem pieces were further divided into six pieces (1cm in length) by cutting with a scalpel on a sterile glass plate. The two leaves were each divided into four smaller pieces (ca. 0.5 cm^2^) and plated together in one petri dish. Petri dishes were then checked every two to three days for 30 days under a stereomicroscope for outgrowth of *B*. *bassiana* using the morphological characteristics of the species. For each experimental run (four in total), all seed inoculated plants were checked in addition to two control plants and two sprayed plants per generation (four plants per treatment per experimental run). We consider a plant colonized by endophytic *B*. *bassiana* if one or more of the plant pieces per plant produces outgrowth of *B*. *bassiana*.

### Statistical analysis

All statistical analyses were done using R [29]. To check whether the formation of alates differed between the treatments, a generalized linear mixed model was used to explain the proportion of winged aphids with treatment and generation as a combined fixed effect and repetition, and individual plants as random factors (*logit* link function). Pairwise comparisons were done using simultaneous tests for general linear hypotheses. Packages used for analysis were multcomp [30] and lme4 [31], while ggplot2 [32] was used for plots.

The statistical analysis of the nymph production data was done using a generalized linear mixed model with treatment and generation as a combined fixed effect and repetition, individual plants, and individual aphids as random factors (*log* link function). A Poisson distribution for the data was used, as the data is count data after a specific time interval (6 days). Pairwise comparisons were done using simultaneous tests for general linear hypotheses. Bonferroni corrected *p* values for reporting significant differences (*p* value < 0.05) were used throughout the analysis. Packages used for analysis were multcomp [30] and lme4 [31], while ggplot2 [32] was used for plots. Calculations of estimates and standard errors were done using the delta method in the package car [33].

## Results

Although we observed a large variance in the proportion of alates between the four repetitions by pooling all treatments (12/119 vs. 51/128 vs. 3/83 vs. 0/86), the alate formation did not differ significantly (*p* values > 0.05, residual *df =* 409, *z* values between -0.31 and 0.70) between treatments (see S1 Document for detailed information on the linear mixed models). Therefore, the analysis of aphid fecundity was carried out without including the fecundity of the alates. The final number of apterous adult aphids analyzed were 63, 62 and 67 in the first-generation and 60, 58 and 39 in the second-generation for leaf, seed and control treatments, respectively (all four repetitions pooled). One adult and no nymphs were found dead inside the clip cages following any of the three treatments. No mycosis was observed in the dead individuals and none of the dead individuals were parasitized.

**Fig 2:**
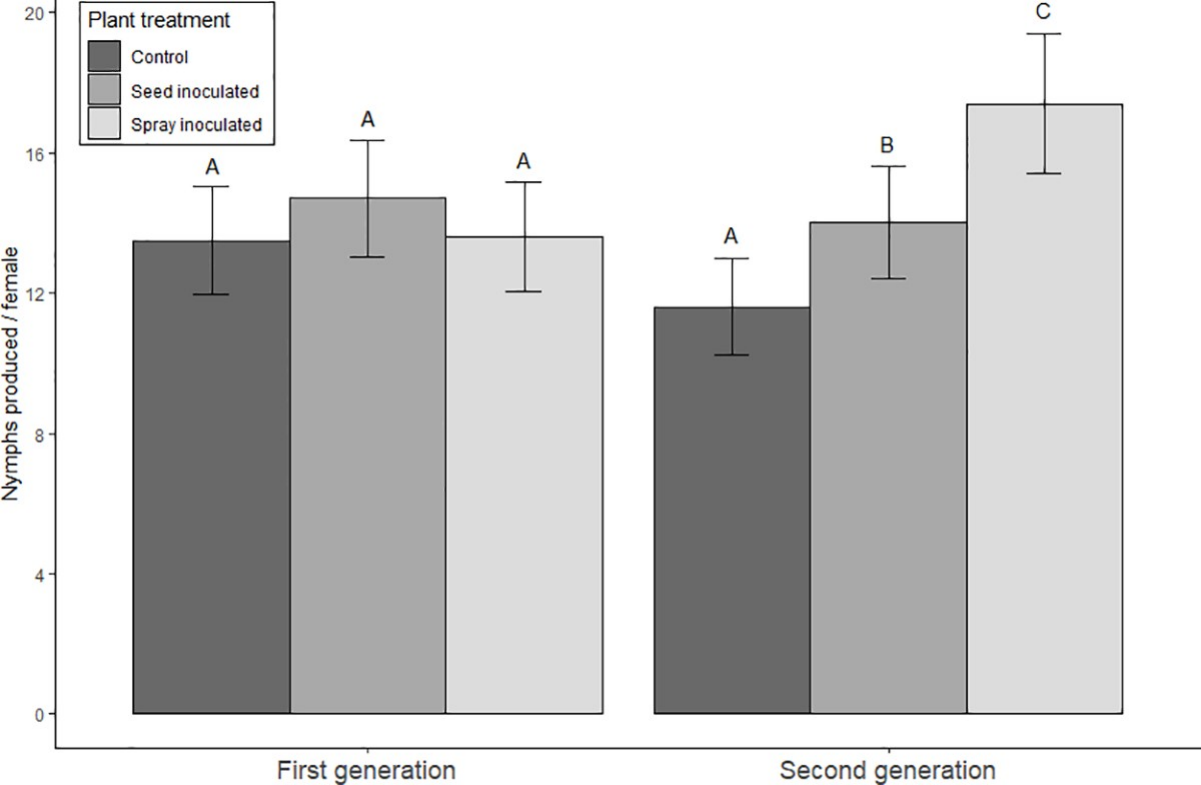
Mean number of nymphs produced by first- and second-generation apterous *A*. *fabae* when exposed to three treatments. The data presented are means from four pooled experimental runs. Error bars represent two times standard error. Different letters indicate statistically significant differences (p value < 0.05), where each generation is analyzed independently.

No statistically significant differences (*p* values > 0.05, residual *df =* 341, z values between -1.67 and -0.25) in fecundity of the first generation of aphids were found between treatments (Fig 2). In the second generation of aphids, however, aphids on *B*. *bassiana* treated plants (sprayed leaves and seed inoculated) produced significantly more nymphs than control aphids (16.9 and 14.7 vs. 12.1; respective *p* values < 0.05, residual *df* = 341, z values -6.58 and -2.94). Furthermore, aphids on leaf inoculated plants produced significantly more nymphs in comparison to the aphids on seed inoculated plants in the second generation (*p* value < 0.05, residual *df* = 341, z value 4.20).

*Beauveria bassiana* was recovered from 26.0% (*n* = 73) of all seed inoculated plants, from 68.8% (*n* = 16) of the sprayed plants, and from one of the control plants (6.3% *n* = 16) (Table 1). In the seed inoculated plants, endophytic *B*. *bassiana* was mostly recovered from roots but was also found in leaves and stems. *Beauveria bassiana* was never recovered from the final rinse water from the surface disinfection process.

**Table 1:** Colonization of *V*. *faba* plants by endophytic *B*. *bassiana* in fecundity tests with *A*. *fabae*.

**Colonized plants (%)**				
**Treatment**	Root	Stem	Leaf	Plant
**Control**	0.0 (-) *n* = 16	0.0 (-) *n* = 16	6.3 (6.2) *n* = 16	**6.3 (6.2) *n* = 16**
**Leaf**	0.0 (0.0) *n* = 16	18.8 (10.5) *n* = 16	68.8 (14.0) *n* = 16	**68.8 (18.1) *n* = 16**
**Seed**	15.1 (4.4) *n* = 73	9.6 (3.6) *n* = 73	9.6 (3.6) *n* = 73	**26.0 (5.9) *n* = 73**
**Colonized plant pieces (20 per plant) (%)**				
**Treatment**	Root (6 per plant)	Stem (6 per plant)	Leaf (8 per plant)	Total (20 per plant)
**Control**	0.0 (-) *n* = 96	0.0 (-) *n* = 96	0.8 (0.9) *n* = 128	0.3 (0.6) *n* = 320
**Leaf**	0.0 (-) *n* = 96	5.2 (2.3) *n* = 96	54.7 (5.1) *n* = 128	23.4 (4.3) *n* = 320
**Seed**	5.5 (1.1) *n* = 438	1.8 (0.6) *n* = 438	1.5 (0.6) *n* = 584	2.8 (0.8) *n* = 1460

The upper part of the table shows the percentage of plants from which *B*. *bassiana* was recovered. The data in the table represent means of pooled data from four experimental runs. The number in brackets denotes the standard error of the estimates. The lower part of table shows the colonization percentage calculated from all plated plant pieces from all treatments (control-, leaf- and seed treatment).

## Discussion

Soil-borne microbes influence plant-mediated effects on arthropods above ground, but the final impacts on insect performance are hard to predict and depend on many variables such as host plant status and the feeding guild of a given herbivore [3]. We hypothesized that *B*. *bassiana* would have a detrimental effect on aphid fecundity. However, the opposite was found, as second-generation aphids on treated plants produced significantly more offspring than aphids on control plants, which is in opposition to previous results with endophytic *B*. *bassiana* (strain GHA) effects on sap-sucking insects [21, 34].

The fact that the differences between treatments in the nymph production of *A*. *fabae* only became apparent in the second generation confirms our notion that two (or multi-) generational effects of endophytic entomopathogenic fungi (EEF) on arthropod populations with fast reproduction and relatively short life spans should be considered in future experimental work. Increased aphid performance has previously been found in plants treated with plant growth-promoting bacteria [35], but positive effects on herbivores by endophytic *B*. *bassiana* have rarely been reported [21]. One study found that endophytic *B*. *bassiana* had some positive effect on the larval biomass of the root-chewing larvae of *Phyllophaga vetula* Horn (Coleoptera: Scarabaeidae) in maize [36]. Using the *B*. *bassiana* GHA strain, some level of positive response (increased reproduction) has been reported previously in cotton aphids after seven days on fungus inoculated plants [37]. Another study found neutral effects on soybean aphid reproduction using endophytic seed treatment with *B*. *bassiana* (GHA), but a significantly increased aphid reproduction on plants inoculated with *Metarhizium brunneum* (strain F52) alone or in combination with *B*. *bassiana* (GHA) [38]. In that study, however, mixed first and second stage nymphs had their subsequent nymph production assessed after 14 days, making any full recording of a second-generation unlikely due to the development time of the aphids.

We attribute the fact that we did not observe a significant effect on the first generation to a general shorter exposure time to the treatments (Fig 1) and a possible transgenerational hormesis effect. When trying to explain the increased performance of the second generation aphids on *B*. *bassiana* inoculated plants observed in this study, we consider three possible explanations: 1) *B*. *bassiana* is improving the quality of the host plants for the aphids. 2) Aphids invest in reproduction because of stress caused by *B*. *bassiana* directly or through upregulation of the plant defense, or 3) *B*. *bassiana* is suppressing the plant defense against aphids via defense pathway cross talk. Several studies have found that EEF benefit plant growth [39–44], and that EEF can translocate nitrogen to their plant host [45]. Specifically, endophytic *B*. *bassiana* has previously been found to increase plant height, number of leaf pairs, fresh root and fresh shoot weight of *V*. *faba* [46]. In this study, they did not investigate the mechanisms behind the growth-promoting effect, but speculated that an increased nutrient uptake and exudation of plant-growth-promoting auxins could be the cause. Furthermore, we used autoclaved soil in our experiments which could have an altered soil chemistry and which does not stay sterile [47]. This could give fungus seed treated plants an advantage over the other treatments against competitive soil-borne pathogens. However, we did not observe any germination issues or stunted growth in any plants. An increase in plant quality, combined with the fact that the quality of the host plant is an important factor in the fecundity of aphids [48, 49], could explain the increased aphid fecundity observed in this study.

Aphids may react to mortality risks by investing in reproduction [50]. While high amounts of stress are harmful to organisms, low amounts can increase some biological processes as seen in many insect taxa [51]. In the present case, the presence of *B*. *bassiana*, or the subsequent stimulation of host plant defenses, may have stressed the aphids, which then invested in reproduction. Aphids on sprayed plants had a higher reproduction than aphids on seed-inoculated and control plants, where the sprayed leaves provided a direct contact between germinating conidia and aphids. The infection potential of germinating conidia could have triggered the aphid stress response of increased production of offspring.

It has previously been hypothesized that endophytic *Metarhizium brunneum* (strain F52) hampers its host plants defense response to an aphid attack by triggering an upregulation of the pathogen defensive pathway via salicylic acid (SA) causing cross-talk between the SA and jasmonic acid (JA) pathway [38, 52]. JA/SA trade-offs has also been shown for beneficial rhizosphere strains of *Pseudomonas* increasing herbivory by the cabbage looper [53]. Furthermore, endophytic *B*. *bassiana* in maize has been shown to initiate plant defense pathways similar to that of a pathogen attack [54]. This could be a general response of EEF colonization, thereby, also a possible explanation of our results. This hypothesis is highly relevant for the understanding of the complex multi-trophic interactions between fungi, plants, and herbivores and requires further investigation on a molecular level.

We recovered *B*. *bassiana* from one-quarter of the seed inoculated plants and from two-thirds of the sprayed plants via re-isolation using an efficacious surface disinfection method. This shows that *B*. *bassiana* was able to grow as an endophyte in *V*. *faba* in our experimental set-up and also shows that it developed as an endophyte in the spray treatment over the duration of the fecundity experiment (as we sprayed the plants only two hours before placing the aphids). We noted that, when recovered from a plant, it was very rarely more than one of the six pieces per plant part that supported outgrowth of the fungus. This is also reflected in the large proportion of plants from which *B*. *bassiana* was recovered compared to that of the plated plant pieces (Table 1). This highlights an issue with the re-isolation method, where only a minor part of the whole plant is sampled, which subsequently complicates the quantification of endophytic colonization. Moreover, growth competition between *B*. *bassiana* and faster growing endophytes may diminish colonization results further [46], as we did observe other fungi growing in the plates, especially from the roots. A study has also shown that the amount of *B*. *bassiana* in the leaves following seed inoculation diminishes over a 28 days period [55], a fact that would cause us to underestimate the presence of *B*. *bassiana* during the experiments in our sampling and subsequent observation of plated plant pieces. Despite the caveats of endophyte detection, it does not make the actual symbiotic interaction between plants and entomopathogenic fungi less relevant. This was argued previously by Tall and Meyling (43), who found a growth-promoting effect of *B*. *bassiana* (strain GHA) seed treatment in maize, but who was unable to recover endophytic *B*. *bassiana* via PCR. Still, more knowledge on the relationship between fungal colonization (recovery) and herbivore effects is needed.

As shown here, prediction of the outcome of a microbe-plant-insect interaction is difficult as it relies on a myriad of factors. In order to gain further knowledge on how EEF influences arthropods, it is important to consider longer-term effects, when possible (e.g., several generations). In this study, we showed that a longer exposure time (first vs. second-generation) led to an increased effect of an EEF on *A*. *fabae*. In addition to this, the observed effect on aphid reproduction was positive. This stands in contrast to the negative effects found by the majority of studies done on EEF effects on one generation of various herbivores [13]. This is one of very few reports of a positive effect on herbivore reproduction by an EEF (and the first clear report in regards to endophytic *B*. *bassiana)*, and while we have suggested possible mechanisms responsible for this positive effect, there is a general lack of knowledge of the mechanisms behind EEF effects on different pests and beneficial insects.

The complexity of multi-trophic interactions is also important to consider in a biocontrol setting. It is necessary to take into account that treating crops with EEF might not have a controlling effect on a given pest, but might, as shown in this study, actually increase the performance of a given herbivore. Likewise, natural enemies might also be influenced in unpredictable ways by EEF. It is, therefore, important to investigate potential synergistic or antagonistic effects between EEF and other biocontrol agents.

## Supporting information

S1 Dataset Data on nymph production used for analysis. (XLSX)

S1 Document Additional information on the linear mixed models used. (DOCX)
