# Supplementary material for: Increased fecundity of Aphis fabae on Vicia faba plants following seed or leaf inoculation with the entomopathogenic fungus Beauveria bassiana
Source: PLoS One. 2019 Oct 7;14(10):e0223616. doi: 10.1371/journal.pone.0223616 (PMC6779261; doi:10.1371/journal.pone.0223616)
Supplement: S1 Document — (DOCX) [file pone.0223616.s002.docx]

**Supporting information S1 Document.** Additional information on the linear mixed models used.

**Model on alate formation.**

Formula used in R software. Generalized linear mixed model (glmer): Alates ~ treatmentgeneration -1 + (1 | repetition) + (1 | plant)

Fixed effects

| **Treatment and Generation** | **β estimates** | **Std. Error** | **z value** |
| --- | --- | --- | --- |
| Control 1^ST^ generation | -11.504 | 2.598 | -4.428 |
| Seed 1^st^ generation | -11.589 | 2.624 | -4.416 |
| Spray 1^st^ generation | -12.703 | 3.581 | -3.547 |
| Control 2^nd^ generation | -8.373 | 1.811 | -4.624 |
| Seed 2^nd^ generation | -9.728 | 2.020 | -4.815 |
| Spray 2^nd^ generation | -8.691 | 1.483 | -5.861 |

Random effects

| **Group** | **Variance** | **Std. Dev.** |
| --- | --- | --- |
| Individual plant (Plant ID) | 149.106 | 12.211 |
| Repetition | 1.966 | 1.402 |

**Model on nymph production**

Formula used in R software. Generalized linear mixed model (glmer): Nymphs ~ treatmentgeneration -1 + (1 | repetition) + (1 | plant) + (1 | aphid ID)

Fixed effects

| **Treatment and Generation** | **β estimates** | **Std. Error** | **z value** |
| --- | --- | --- | --- |
| Control 1^ST^ generation | 2.6002 | 0.1138 | 22.84 |
| Seed 1^st^ generation | 2.6867 | 0.1138 | 23.60 |
| Spray 1^st^ generation | 2.6133 | 0.1140 | 22.92 |
| Control 2^nd^ generation | 2.4495 | 0.1191 | 20.57 |
| Seed 2^nd^ generation | 2.6369 | 0.1146 | 23.01 |
| Spray 2^nd^ generation | 2.8588 | 0.1136 | 25.16 |

Random effects

| **Group** | **Variance** | **Std. Dev.** |
| --- | --- | --- |
| Individual plant (plant ID) | 8.849e-03 | 1.727e-05 |
| Individual aphid (aphid ID) | 2.981e-10 | 9.407e-02 |
| Repetition | 4.637e-02 | 2.153e-01 |
